# Association Between the High Stress Hyperglycemia Ratio and Outcomes of Mechanical Thrombectomy: A Systematic Review and Meta-Analysis

**DOI:** 10.7759/cureus.91024

**Published:** 2025-08-26

**Authors:** Aliu O Yakubu, Moses G Effiong, Oreoluwa Morakinyo, Tosin T Fakiyesi, Gregory I Atafo

**Affiliations:** 1 Neurology, University of Texas Medical Branch, Galveston, USA; 2 Public Health, Glasgow Caledonian University, Glasgow, GBR; 3 General Practice, Shirley Oaks Hospital, Croydon, GBR; 4 Internal Medicine, Korle Bu Teaching Hospital, Accra, GHA

**Keywords:** acute ischemic stroke, hyperglycemia, mechanical thrombectomy, stress hyperglycemia ratio, stroke

## Abstract

Stroke is a major cause of global death and disability, with mechanical thrombectomy (MT) as the gold standard treatment for large vessel occlusion. The stress hyperglycemia ratio (SHR), which accounts for long-term glycemic control via glycated hemoglobin (HbA1c), is a promising predictor of stroke outcomes. However, its prognostic value in acute ischemic stroke (AIS) patients treated with MT has not been systematically evaluated. We conducted a meta-analysis to assess the association between SHR and outcomes after MT. PubMed, Embase, Cochrane Library, and Google Scholar were searched. Analyzed outcomes included functional outcomes, mortality, early neurological deterioration (END), and symptomatic intracranial hemorrhage (sICH). Data were pooled using random-effects models, with heterogeneity assessed by I² statistics. High SHR was significantly associated with increased 90-day poor outcomes (OR=2.98; 95% CI: 1.89-4.71; p<0.0001), low 90-day good outcomes (OR=0.43; 95% CI: 0.32-0.59; p<0.0001), increased 90-day mortality (OR=2.06; 95% CI: 1.37-3.11; p=0.0005), higher risk of END (OR=6.27; 95% CI: 3.22-12.24; p<0.0001), and sICH (OR=2.53; 95% CI: 1.73-3.70; p<0.0001). Elevated SHR predicts poor outcomes, higher mortality, and more complications in AIS patients undergoing MT, highlighting its importance in stroke management.

## Introduction and background

Stroke remains a leading cause of death and long-term disability worldwide. According to the World Health Organization, stroke accounted for approximately 10% of all global deaths in 2021, making it the third leading cause of death that year [1]. Acute ischemic stroke (AIS) resulting from large vessel occlusion (LVO) represents one of the most severe subtypes and is associated with poor outcomes if not promptly treated [2]. MT has emerged as the gold standard intervention for AIS due to LVO, offering superior recanalization and functional outcomes compared to medical therapy alone [2-4].

Hyperglycemia is a well-recognized response to acute physiological stress, including stroke, and is associated with increased mortality and worse neurological outcomes [5,6]. This stress-induced hyperglycemia results from activation of the hypothalamic-pituitary-adrenal axis and sympathetic nervous system, leading to elevated cortisol and catecholamines [5]. In the context of stroke, hyperglycemia promotes a pro-inflammatory and pro-oxidative environment, leading to blood-brain barrier (BBB) disruption through oxidative stress-induced activation of matrix metalloproteinases (MMPs) and degradation of endothelial tight junction proteins. These processes, together with lactic acidosis and direct neuronal injury, exacerbate ischemic damage and impair recovery [7-9]. These effects are relevant in patients undergoing reperfusion therapies such as MT, as they affect the efficacy of thrombolysis and thrombectomy by reducing fibrinolytic activity, contributing to reperfusion injury and hemorrhagic complications [8,10].

Despite its clinical importance, there is no universally accepted guideline for defining stress hyperglycemia in AIS. Traditional markers such as admission blood glucose or fasting plasma glucose do not account for baseline glycemic control, particularly in patients with pre-existing diabetes [11]. Many studies have relied on admission, random, or fasting glucose levels to define stress hyperglycemia. According to the American Diabetes Association (ADA), hyperglycemia in hospitalized individuals is defined as blood glucose >140 mg/dL (>7.8 mmol/L) [11]. However, this definition is limited to individuals with known diabetes, as it overlooks variations in chronic glycemic control.

To address these limitations, the stress hyperglycemia ratio (SHR), calculated by dividing admission glucose by glycated hemoglobin (HbA1c), has been proposed as a more accurate indicator of acute glycemic dysregulation relative to chronic glucose status [12]. Previous observational studies have shown that elevated SHR is associated with increased mortality, poorer functional outcomes, and a higher risk of complications in AIS patients [13,14]. This relationship may be more relevant in patients undergoing MT, who typically present with LVO and are subjected to greater procedural and metabolic stress. By accounting for both acute and chronic glycemic states, SHR may serve as a valuable biomarker for identifying MT-treated patients at higher risk of poor prognosis, early neurological deterioration (END), or hemorrhagic complications. Such risk stratification could guide closer hemodynamic and metabolic monitoring and support early implementation of complication-prevention strategies in high-risk patients.

Previous meta-analyses have examined the prognostic value of SHR in broader AIS populations, often combining patients treated with intravenous thrombolysis (IVT), MT, or conservative management [15,16]. However, to our knowledge, no prior meta-analysis has focused on AIS patients treated with MT only, nor specifically assessed the prognostic value of SHR in this high-risk subgroup. A further limitation of these studies is the predominant use of SHR as a continuous variable, which reduces clinical applicability by lacking standardized thresholds for risk categorization. Categorical stratification is more aligned with real-world bedside decision-making, where clinicians must quickly assess risk and guide management accordingly. Unlike continuous variables, categorical cut-offs provide clearer thresholds that facilitate risk stratification, prognostication, and protocol-driven interventions within acute stroke workflows, thereby enhancing their clinical utility. Additionally, pooling heterogeneous treatment groups may obscure risks that are specific to MT, such as reperfusion injury or symptomatic intracranial hemorrhage (sICH) associated with catheter-based interventions.

This study addresses these gaps through a systematic review and meta-analysis focused solely on AIS patients undergoing MT. To enhance clinical relevance, we synthesized adjusted effect estimates from studies that used SHR as a categorical predictor of clinically relevant outcomes, providing more practical utility for bedside risk assessment than continuous SHR values. Our objective was to evaluate the prognostic significance of SHR in relation to functional recovery, mortality, and MT-related complications, and to assess its potential utility as a risk stratification tool in thrombectomy-based stroke care.

## Review

Methods

The study adhered to the guidelines set by the Preferred Reporting Items for Systematic Reviews and Meta-Analyses (PRISMA) [17]. It was registered with the International Prospective Register of Systematic Reviews (PROSPERO) under the identifier CRD420251002849. This systematic review and meta-analysis aimed to evaluate the association between SHR and functional and clinical outcomes following MT. Additionally, the study aimed to investigate the relationship between SHR and early neurological deterioration and complications such as sICH.

Search Strategy

A systematic search of eligible studies was conducted in several databases, including PubMed, Embase, the Cochrane Library, and Google Scholar, from inception to March 15, 2025. Only studies published in English were included. The search terms utilized included ("stress hyperglycemia" OR "stress hyperglycemia ratio") AND ("mechanical thrombectomy" OR "endovascular thrombectomy" OR "intra-arterial therapy"). The search was supplemented by reviewing all the articles that cited the papers included in the review ("snowballing") and checking the reference list. This was exported to Rayyan for deduplication, screening, and study selection.

Eligibility Criteria

Studies were selected according to the PICO framework. The population included adults aged 18 years or older with AIS due to LVO, treated with MT, with or without pre-existing diabetes. The intervention of interest was the assessment of the SHR. The comparator was defined as a lower SHR category (Q1, T1, or low SHR group) versus a higher SHR category (Q4, T3, or high SHR group), according to each study’s reported stratification. To address heterogeneity in categorization schemes (quartiles, tertiles, or dichotomized groups), we pooled effect estimates by comparing the most extreme categories (highest vs. lowest SHR) within each study. This harmonization allowed consistent synthesis of risk estimates while respecting study-specific definitions. The primary outcomes were 90-day good outcome (mRS 0-2), poor outcome (mRS 3-6), and mortality. Secondary outcomes included END and sICH. Eligible studies were either observational or interventional and were required to report adjusted odds ratios (ORs) with 95% confidence intervals (CIs) for the association between SHR (as a categorical variable) and at least one outcome of interest. Studies that reported SHR only as a continuous variable were excluded to ensure consistency in the pooled effect measures. Additional exclusion criteria included abstracts, case reports, cross-sectional studies, literature reviews, non-English language publications, and studies focused on thrombolysis only or populations not treated with MT.

Data Extraction and Quality Assessment

Data extraction was conducted by one reviewer and cross-verified by another reviewer. Extracted data included study characteristics, patient demographics, comorbidities, SHR definition, cutoff values, glucose measurement timing, and ORs of clinical outcomes. The quality of the included studies was assessed using the Newcastle-Ottawa Scale (NOS) [18], which evaluates studies based on selection, comparability, and outcome. Studies with a score of more than six stars were considered high quality. Cochrane risk of bias for randomized controlled trials [19]. Any discrepancies were resolved through consultation with another reviewer.

Statistical Analysis

The statistical analysis was performed using R (R Development Core Team, Vienna, Austria) software. The adjusted ORs with 95% CIs for high/low SHR, quartile (Q4 vs. Q1), and tertile (T3 vs. T1) groupings were pooled using the DerSimonian-Laird random-effects model to account for heterogeneity. For each study, the logarithm of the OR (logOR) and its standard error (SE(logOR)) were computed. A p-value of < 0.05 was considered statistically significant. Heterogeneity between studies was assessed using the Cochrane Q test, with p < 0.1 or I² > 50% indicating significant heterogeneity. 

Results

**Figure 1:**
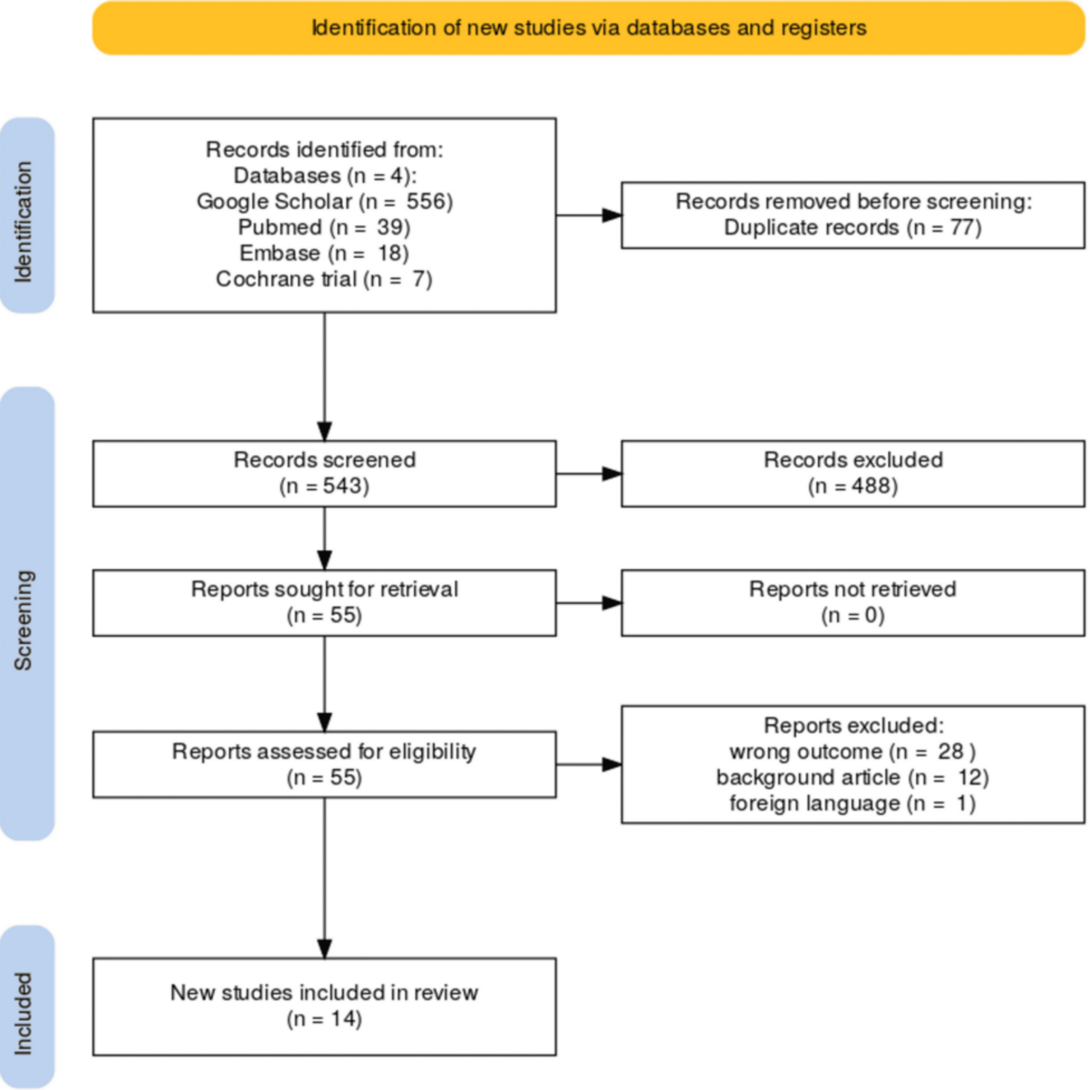
PRISMA flow of the included studies PRISMA: Preferred Reporting Items for Systematic Reviews and Meta-Analyses

The initial search identified 630 articles, of which 14 studies met the inclusion criteria and were included in the systematic review [13,14,20-31]. The PRISMA flow diagram illustrates the study selection process (Figure 1). 

Table 1 and the Appendix present the data summary of the included studies. Among these are 11 retrospective studies, two prospective studies, and one randomized controlled clinical trial. Of the 14 studies, 12 were conducted in China. The studies were carried out between 2018 and 2024, with a total sample size of 5,803 participants, 3,741 (64.5%) of whom were male. The mean age of participants was 68.1 years. The stroke subtypes were as follows: large artery atherosclerosis accounted for 2,346 cases (40.4%), cardioembolism for 2,732 cases (47.1%), and other or undetermined stroke etiologies made up 800 cases (13.8%). The most common comorbidities were hypertension (64.1%) and atrial fibrillation (28.3%). The definition of SHR commonly used across the studies was fasting plasma glucose (FPG; mmol/L)/HbA1c (%) in seven studies. Regarding SHR groupings, four studies used the low/high SHR classification, six used quartiles, and three used tertiles. All included cohort studies were assessed as high quality using the NOS (Appendix), while the randomized controlled trial demonstrated some concerns regarding risk of bias.

**Table 1:** Data summary of the included studies LAA: Large Artery Atherosclerosis, CE: Cardioembolic Stroke, ODE: Other Determined Etiologies, SUE: Stroke of Undetermined Etiology, NIHSS: National Institutes of Health Stroke Scale, SHR: Stress Hyperglycaemic Ratio, GAR: Glucose-to-Glycated Hemoglobin ratio, Q: Quartile, T: Tertile, FPG: Fasting Plasma Glucose, HbA1c: Hemoglobin A1c, NS: Not Stated, NOS: Newcastle–Ottawa Scale

First Author, Year, Country	Sample Size	Male, n (%)	Stroke Subtypes (TOAST Criteria)	Mean NIH Score at Admission/Discharge	Definition of SHR (Formula Used)	SHR Group	SHR Cutoff Values Used	Blood Glucose Measurement Timing	NOS
Dai et al., 2023 (China) [13]	559	357 (63.86%)	LAA-271 (48.48%). CE-231 231 (41.32%). Others: -57 (10.19%)	16/NS	FPG/HbA1c (%)	High/Low	NS	NS	9/9
Merlino et al., 2021 (Italy) [14]	204	100 (49.02%)	LAA: 24 (11.7%). CE: 110 (53.9%). ODE: 6 (2.94%). SUE: 64 (31.4%)	16.5/5.75	FPG (mg/dL)/HbA1c (%)	Quartile	Q1: 15.1 (13.6-15.7). Q2: 17.6 (16.9-18.1). Q3: 19.8 (18.9-20.8). Q4: 25.4 (23.1-28.4)	24 hrs	9/9
Yang et al., 2024 (China) [20]	553	368 (66.54)	LAA: 323 (58.4%). CE: 189 (34.2%). ODE: 20 (3.61%). SUE: 21 (3.79%)	15/6.75	FPG (mmol/L)/HbA1c (%)	Quartile	(1st quartile): GAR < 0.93 (2nd quartile): 0.93 ≤ GAR < 1.13 (3rd quartile): 1.13 ≤ GAR < 1.39 (4th quartile): GAR ≥ 1.39	24 hrs	8/9
Zhang et al., 2022 (China) [21]	408	265 (65%)	LAA: 172 (42.15%). CE: 183 (44.85%). ODE: 32 (7.84%). SUE: 21 (5.15%)	14.5/6.5	FPG (mmol/L)/HbA1c (%)	Quartile	NS	24 hrs	8/9
Wang et al., 2018 (China) [22]	204	196 (61.05%)	NS	16/NS	SHR = Admission Blood Glucose/Estimated Average Blood Glucose (calculated from HbA1c) Formula for Estimated Average Glucose (EAG): EAG = (1.59 × HbA1c) – 2.59	Quartile	Q1: Median SHR 0.81 (IQR 0.70–0.87). Q2: Median SHR 1.05 (IQR 0.97–1.10). Q3: Median SHR 1.40 (IQR 1.26–1.58)	NS	8/9
Duan et al., 2023 (China) [23]	576	328 (56.94%)	LAA: 307 (53.29%). CE: 171 (29.68%). ODE: 98 (17.01%)	14.91/9.92	FPG (mmol/L)/HbA1c (%)	Quartile	DM Group: Q1: ≤ 0.986 Q2: 0.986 < GAR ≤ 1.187 Q3: 1.187 < GAR ≤ 1.497 Q4: > 1.497. Non-DM Group: Q1: ≤ 0.93 Q2: 0.93 < GAR ≤ 1.075 Q3: 1.075 < GAR ≤ 1.331 Q4: > 1.331​	24 hrs	9/9
Merlino et al., 2024 (Italy) [24]	691	336 (48.63%)	LAA: 123 (17.80%). CE: 333 (48.19%). ODE: 45 (6.51%). SUE: 190 (27.50%)	16/NS	FPG (mg/dL)/HbA1c (%)	Quartile	Q1: < 17.0. Q2: 17.0–20.0. Q3: 20.1–24.8. Q4: > 24.8​	24 hrs	8/9
Chen et al., 2019 (China) [25]	160	108 (67.50%)	LAA: 79 (49.4%). CE: 60 (37.5%). ODE or SUE: 21 (13.1%)​	15/NS	SHR = Admission Blood Glucose/Estimated Average Blood Glucose (calculated from HbA1c) Formula for Estimated Average Glucose (EAG): EAG = (1.59 × HbA1c) – 2.59	High/Low	0.96	48 hrs	8/9
Sun et al., 2023 (China) [26]	423	250 (59.10%)	LAA: 119 (28.1). CE: 257 (60.8). ODE or SUE: 47(11.1)	14/NS	FBG/A1c-derived average glucose (ADAG)	High/Low	0.89	24 hrs	9/9
Peng et al., 2024 (China) [27]	250	188 (75.20%)	LAA: 167 (66.8%). CE: 66 (26.4%). ODE or SUE: 17 (6.8%)	23/NS	Glucose (mmol/L)/HbA1C (%)	Tertile	T1 (≤ 1.11). T2 (1.12–1.36). T3 (≥ 1.37)	NS	8/9
Shi et al., 2024 (China) [28]	285	154 (54.04%)	LAA: 89 (30.9%). CE: 179 (59.3%). Other causes: 28 (9.8%)	17/NS	SHR = Admission Glucose (mmol/L) / (1.59 × HbA1C - 2.59)	Tertile	T1: ≤0.94. T2: 0.95–1.15. T3: ≥1.16	NS	9/9
Liu et al., 2022 (China) [29]	739	471 (63.7%)	LAA: 332 (44.9%). CE: 349 (47.2%). ODE: 22 (3.0%). SUE: 36 (4.9%)	14/NS	SHR = FBG/Chronic Glycemia Ratio Chronic Glucose Level (mg/dL) = 28.7 × HbA1c (%) - 46.7	High/Low	0.96	24 hrs	9/9
Wang et al., 2023 (China) [30]	209	114 (54.5%)	LAA: 94 (44.98%). CE: 96 (45.93%). Other causes: 19 (9.09%)	14.33/NS	FPG (mmol/L)/HbA1c (%)	Quartile	Non-diabetic: Q1 (≤1.12). Q2 (1.13–1.29). Q3 (≥1.30) Diabetic: Q1 (≤1.56) Q2 (1.57–1.79) Q3 (≥1.80)	24 hrs	7/9
Peng et al., 2023 (China) [31]	542	306 (56.5%)	LAA: 246 (45.4%). CE: 240 (44.3%). Other causes: 56 (10.3%)	16/NS	Admission Glucose (mmol/L)/HbA1C (%)	Tertile	T1: ≤1.07. T2: 1.08–1.29. T3: ≥1.30	NS	-

Primary and Secondary Clinical Outcomes

**Figure 2:**
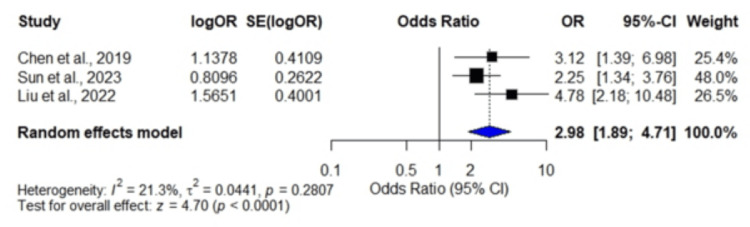
Forest plots showing odds ratios of 90-day poor outcome Data are derived from Chen et al. [25], Sun et al. [26], and Liu et al. [29].

Ninety-day poor outcome (mRS: 3-6): A total of three studies [25,26,29] involving 1,322 patients demonstrated a pooled OR of 2.98 (95% CI: 1.89, 4.71). A high SHR was significantly associated with an increased risk of 90-day poor functional outcome (mRS: 3-6) (p < 0.0001) with low heterogeneity (I² = 21.3%, p = 0.2807) (Figure 2).

**Figure 3:**
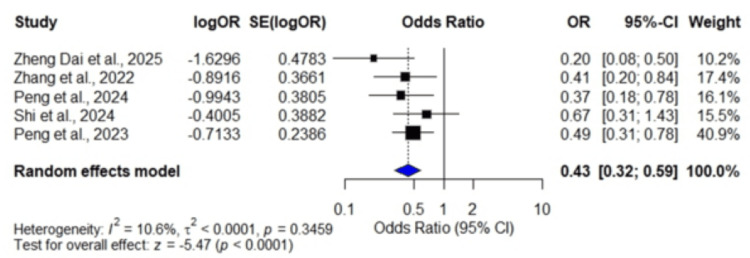
Forest plot showing odds ratios for 90-day good functional outcome Data were extracted from five studies: Dai et al., 2025 [13]; Zhang et al., 2022 [21]; Peng et al., 2024 [27]; Shi et al., 2024 [28]; and Peng et al., 2023 [31].

Ninety-day good functional outcome (mRS: 0-2): Five studies [13,21,27,28,31] involving 2,044 patients demonstrated a pooled OR of 0.43 (95% CI: 0.32, 0.59; p < 0.0001), indicating that high SHR was significantly associated with lower rates of achieving 90-day good functional outcome (mRS: 0-2). Low heterogeneity was observed among the included studies (I² = 10.6%, p = 0.3459) (Figure 3).

**Figure 4:**
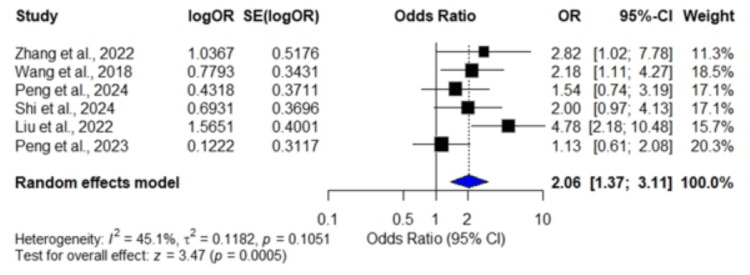
Forest plot showing odds ratios for 90-day mortality Data were extracted from six studies: Zhang et al., 2022 [21]; Wang et al., 2018 [22]; Peng et al., 2024 [27]; Shi et al., 2024 [28]; Liu et al., 2022 [29]; and Peng et al., 2023 [31].

Ninety-day mortality: Six studies [21,22,27,28,29,31] demonstrated a pooled OR of 2.06 (95% CI: 1.37, 3.11; p = 0.0005), indicating that a high SHR was significantly associated with an increased risk of 90-day mortality (p = 0.0005). Moderate heterogeneity was observed among the included studies (I² = 45.1%, p = 0.1051; Figure 4).

Safety

**Figure 5:**
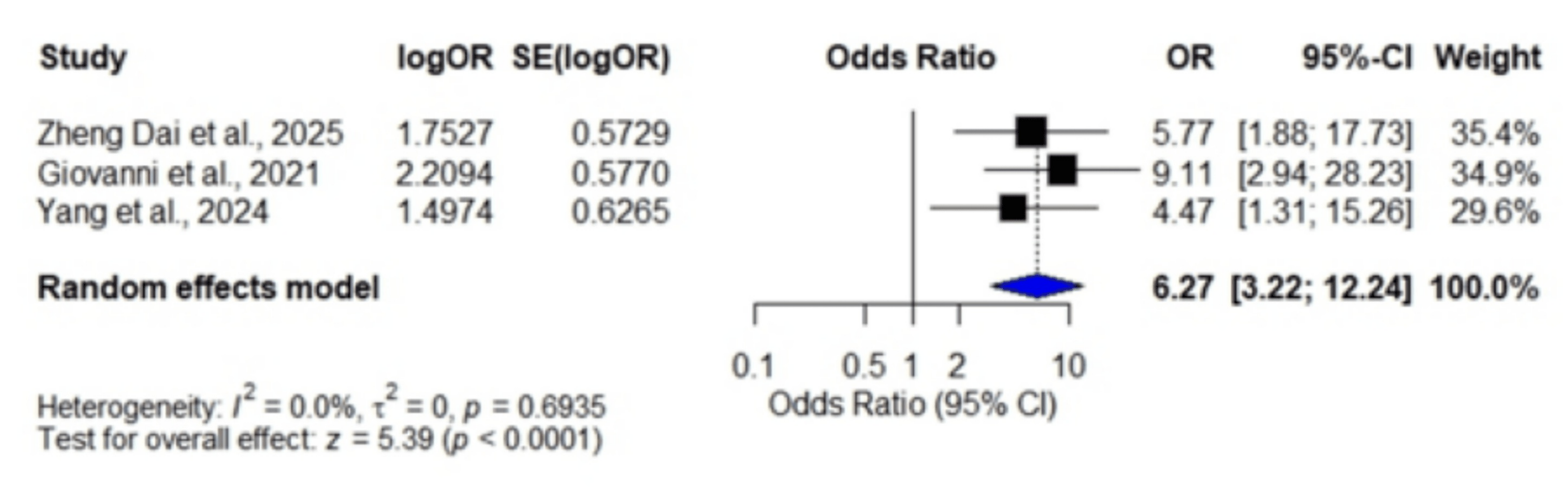
Forest plot showing odds ratios for early neurological deterioration Data were obtained from three studies: Dai et al., 2025 [13]; Giovanni et al., 2021 [14]; and Yang et al., 2024 [20].

Early neurological deterioration: A total of three studies [13,14,20] involving 1,316 patients demonstrated a pooled OR of 6.27 (95% CI: 3.22, 12.24), indicating that a high SHR was significantly associated with an increased risk of early neurological deterioration (p < 0.0001). No heterogeneity was observed among the included studies (I² = 0.0%, p = 0.6935) (Figure 5).

**Figure 6:**
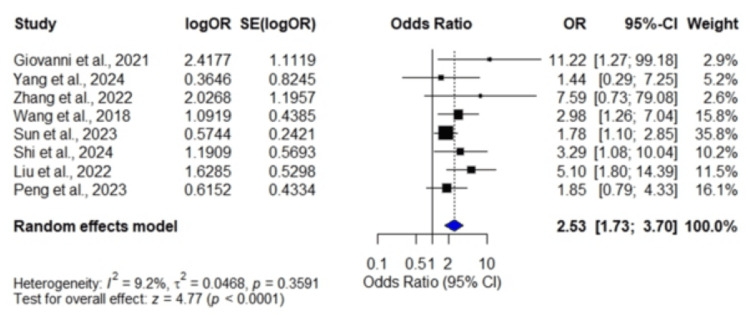
Forest plot showing odds ratios for symptomatic intracranial hemorrhage Data were obtained from eight studies: Giovanni et al., 2021 [14]; Yang et al., 2024 [20]; Zhang et al., 2022 [21]; Wang et al., 2018 [22]; Sun et al., 2023 [26]; Shi et al., 2024 [28]; Liu et al., 2022 [29]; and Peng et al., 2023 [31].

Symptomatic intracranial hemorrhage (sICH): A total of eight studies [14,20,21,22,26,28,29,31] involving 3,475 patients demonstrated a pooled OR of 2.53 (95% CI: 1.73, 3.70; p < 0.0001), indicating that a high-stress glycemic ratio was significantly associated with an increased risk of sICH. Low heterogeneity was observed among the included studies (I² = 9.2%, p = 0.3591) (Figure 6).

Discussion

This meta-analysis evaluated the association between SHR and clinical outcomes in patients with AIS undergoing MT for LVO. By analyzing data from 5,803 patients across diverse populations, our study provides robust evidence that elevated SHR is significantly associated with a higher risk of adverse outcomes, including poor functional recovery, increased 90-day mortality, END, and sICH.

Specifically, patients with elevated SHR had more than twice the odds of mortality at 90 days compared to those with lower SHR, underscoring its value as an independent predictor of post-stroke survival. This aligns with prior research identifying stress hyperglycemia as a prognostic factor in AIS populations [21-23]. For instance, Chen et al. demonstrated that SHR predicted both in-hospital and ICU mortality among critically ill coronary heart disease patients, further supporting the generalizability of SHR as a mortality marker [32].

In terms of functional outcomes, our findings indicate that high SHR is strongly predictive of poor neurological recovery. Patients with elevated SHR had significantly higher odds of poor functional outcome (mRS: 3-6) at 90 days post-stroke (OR = 2.98; 95% CI: 1.89-4.71; p < 0.0001). These findings are consistent with prior meta-analyses, including [15], which demonstrated that AIS patients with poor prognoses had higher SHR values than those with favorable outcomes (SMD = 0.56, 95% CI: 0.37-0.75; p < 0.001). Our studies focusing only on MT patients enhance the clinical relevance of SHR in predicting the outcome of endovascular therapy, a population in which prognostic markers are critically needed to guide post-procedural care and stratify risk.

Pathophysiological Mechanisms of Hyperglycemia-Induced Injury

Endocrine and inflammatory triggers: Stress hyperglycemia in AIS arises from a multifactorial process involving neuroendocrine and inflammatory responses. Counter-regulatory hormones, such as cortisol, catecholamines, and pro-inflammatory cytokines, drive hepatic gluconeogenesis and worsen insulin resistance, fueling hyperglycemia during acute illness [33,34]. This maladaptive response magnifies metabolic stress, amplifying downstream injury pathways in the ischemic brain.

Oxidative stress and BBB breakdown: Oxidative stress is a major pathway linking hyperglycemia to secondary brain injury. Mitochondrial dysfunction during ischemia leads to excessive reactive oxygen species (ROS) production, which is exacerbated under hyperglycemic conditions [10]. Activated leukocytes and microglia further amplify oxidative damage, increasing lactic acidosis and accelerating neuronal death.

Inflammatory mediators with excessive MMP-9 break down the vascular basal lamina and tight junction proteins, resulting in BBB disruption [35,36]. Experimental studies implicated hypoxia inducible factor-1 (HIF‑1α) and vascular endothelial growth factor (VEGF) in the breakdown of the BBB, infarct size, hemorrhagic transformation, and neurologic deficits [36]. The suppression of this endothelial HIF-1α through the activity of endothelial HIF-1α knockout lowers the risk of these poor outcomes. Glycemic control with insulin lowers HIF-1α upregulation, leading to decreased BBB permeability and smaller infarctions. Consistent with these findings, our analysis showed that high SHR was associated with an increased risk of sICH, likely reflecting weakened microvascular integrity. Additionally, hyperglycemia stimulates the extracellular release of high mobility group box 1 (HMGB1), which functions as a damage-associated molecular pattern (DAMP) molecule. Extracellular HMGB1 activates Toll-like receptor 4 (TLR4) and receptor for advanced glycation end products (RAGE) pathways, amplifying neuroinflammation, promoting BBB disruption, and exacerbating infarct size, neurological deficits, and cerebral edema [37]. Inhibition of HMGB1 with glycyrrhizin has been shown to preserve tight junction integrity, suggesting a potential therapeutic strategy for hyperglycemic stroke.

Metabolic dysfunction and neuronal death: Hyperglycemia worsens ischemic energy failure by promoting anaerobic glycolysis, lactate accumulation, and tissue acidosis [10]. Under normoglycemic conditions, high-energy phosphate metabolites - primarily ATP - recover rapidly after ischemia, reflecting the metabolic resilience and functional stability of mitochondria [38]. In contrast, hyperglycemia exacerbates cortical acidosis and impairs mitochondrial function, resulting in delayed restoration of both high-energy phosphates and intracellular pH. This acidic pH triggers enzymatic processes that promote cell death. Furthermore, hyperglycemia intensifies excitotoxic neuronal injury by increasing ischemia-induced glutamate release and impairing its clearance in high-glucose environments [12]. Elevated extracellular glutamate overstimulates NMDA and AMPA receptors, leading to excessive calcium influx, which triggers mitochondrial dysfunction, heightened ROS production, and activation of apoptotic pathways, ultimately resulting in neuronal damage and death [39].

Coagulation and microvascular injury: Hyperglycemia also promotes a procoagulable and microcirculatory-impaired state. Elevated plasminogen activator inhibitor-1 (PAI-1) and reduced tissue plasminogen activator (tPA) activity inhibit fibrinolysis and favor thrombosis [40]. Hyperglycemia also causes endothelial dysfunction through inhibition of nitric oxide (NO) bioavailability, which promotes leukocyte adhesion to blood vessel walls [41]. The net result is propensity for microvascular thromboses and impaired microvascular reperfusion, even despite successful endovascular recanalization of the primary occlusion. Hyperglycemia contributes to endothelial dysfunction through endothelial NO synthase uncoupling, resulting in reduced NO production and impaired reperfusion. It also promotes the accumulation of ROS via NADPH oxidase (NOX) activation, thereby increasing BBB permeability in both in vitro and in vivo models [42]. These mechanisms align with our findings that elevated SHR was associated with higher odds of sICH after MT.

Clinical Translation

Taken together, these mechanistic insights explain why SHR serves as a powerful biomarker of poor prognosis after MT. Unlike admission glucose, SHR integrates both acute glycemic derangement and chronic glycemic status (via HbA1c), providing a more nuanced measure of stress response in AIS. Conventionally, hyperglycemia in stroke has been defined as glucose ≥10 mmol/L, with guidelines recommending intervention above this threshold [43]. However, the SHINE trial demonstrated that intensive glucose control (4.4-7.2 mmol/L) versus standard control (7.2-10 mmol/L) did not improve outcomes and increased hypoglycemia risk [44]. This underscores that the detrimental effects of stress hyperglycemia are not mediated solely by elevated glucose levels but also by downstream oxidative, metabolic, and vascular pathways.

Recent evidence suggests that SHR outperforms other glycemic indices, including admission glucose, HbA1c, and the glycemic gap, in predicting post-stroke outcomes [45-47]. For instance, SHR ≥1.14 was more strongly associated with poor outcomes than glucose ≥10 mmol/L or GG ≥2.5 mmol/L [45]. Similarly, a nationwide cohort of 167,499 stroke survivors found SHR to have better predictive accuracy for mortality than fasting glucose or HbA1c [46]. Integrating SHR into initial stroke risk assessment could enhance identification of those at risk of developing possible complications who might have been underestimated by blood glucose level only. Liu et al. [29] demonstrated the potential utility of using a glycemia-based ratio (FBG/chronic glycemia, a variant of SHR) in a prognostic nomogram for endovascular stroke therapy. This increased the model's concordance index from 0.811 to 0.841, outperforming models based only on fasting glucose or HbA1c [29].

Building on these findings, our results support incorporating SHR into future predictive models and scoring systems for endovascular stroke management, where it may enhance prognostic accuracy when combined with clinical and imaging factors. SHR may therefore serve as an adjunct marker to guide individualized risk assessment and more tailored clinical decision-making. In patients with elevated SHR, clinicians could anticipate a more complex post-procedural complication, such as increased risks of hemorrhagic transformation or cerebral edema, and adjust their management strategies or intervention thresholds accordingly. Furthermore, SHR can aid in risk-benefit discussions for borderline MT candidates and help set realistic expectations with patients and their families.

Limitations

Several limitations must be acknowledged. Most included studies were retrospective, raising potential selection and reporting biases. Variations in SHR calculation methods, glucose measurement timing, and patient characteristics may have introduced heterogeneity. Restricting inclusion to English-language publications may have excluded relevant data and limited generalizability. Nonetheless, the consistent associations observed across diverse cohorts strengthen the reliability of our conclusions.

## Conclusions

This meta-analysis demonstrated that high SHR is a significant predictor of poor outcomes following MT in patients with AIS. These findings support SHR as a valuable prognostic marker that could be incorporated into existing stroke prognostic models to guide patient monitoring and post-thrombectomy care. Importantly, SHR should complement rather than replace established clinical and imaging predictors. Prospective, multicenter studies are warranted to define standardized SHR cutoffs and to evaluate the utility of SHR-guided interventions in improving stroke recovery.

## Appendices

**Table 2:** Database search strategy

Database	Search Strategy	Result Retrieved
Pubmed	1. ("stress hyperglycemia"[MeSH Terms] OR "stress hyperglycemia ratio") 2. ("mechanical thrombectomy"[MeSH Terms] OR "endovascular thrombectomy" OR "intra-arterial therapy") 3. 1 AND 2	39
Embase	1. ('stress hyperglycemia'/exp OR 'stress hyperglycemia ratio') 2. ('mechanical thrombectomy'/exp OR 'endovascular thrombectomy' OR 'intra-arterial therapy') 3. 1 AND 2	18
Cochrane	1. ("stress hyperglycemia" OR "stress hyperglycemia ratio") 2. ("mechanical thrombectomy" OR "endovascular thrombectomy" OR "intra-arterial therapy") 3. 1 AND 2	7
Google scholar	1)"stress hyperglycemia" OR "stress hyperglycemia ratio" 2) "mechanical thrombectomy" OR "endovascular thrombectomy" OR "intra-arterial therapy" 3. 1 AND 2	556

**Table 3:** Quality appraisal of studies based on the Newcastle–Ottawa scale

First Author, Year, Country	1	2	3	4	5	6	7	8	
Yang et al., 2024 (China) (11)	⭐	⭐	⭐	⭐	⭐⭐	⭐	⭐	⭐	9/9
Dai et al., 2023 (China) (13)	⭐	⭐	⭐	⭐	⭐⭐	⭐	⭐	⭐	9/9
Zhang et al., 2022 (China) (22)	⭐	⭐	-	⭐	⭐⭐	⭐	⭐	⭐	8/9
Merlino et al., 2023 (Italy) (23)	⭐	⭐	⭐	⭐	⭐	⭐	⭐	⭐	8/9
Wang et al., 2018 (China) (24)	⭐	⭐	⭐	⭐	⭐	⭐	⭐	⭐	8/9
Duan et al., 2023 (China) (25)	⭐	⭐	⭐	⭐	⭐⭐	⭐	⭐	⭐	9/9
Merlino et al., 2024 (Italy) (26)	⭐	⭐	⭐	⭐	⭐⭐	⭐	⭐	⭐	9/9
Chen et al., 2019 (China)(27)	⭐	⭐	⭐	-	⭐⭐	⭐	⭐	⭐	8/9
Sun et al., 2023 (China) (28)	⭐	⭐	⭐	⭐	⭐⭐	⭐	⭐	⭐	9/9
Peng et al., 2024 (China) (29)	⭐	⭐	⭐	-	⭐⭐	⭐	⭐	⭐	8/9
Shi et al., 2024 (China) (30)	⭐	⭐	⭐	⭐	⭐⭐	⭐	⭐	⭐	9/9
Liu et al., 2022 (China) (31)	⭐	⭐	⭐	⭐	⭐⭐	⭐	⭐	⭐	9/9
Wang et al., 2023 (China) (32)	⭐	⭐	-	⭐	⭐	⭐	⭐	⭐	7/9

**Table 4:** Summary of data on medical comorbidities of the included studies

First Author, Year, Country	Hypertension, n (%)	Coronary Heart Disease, n (%)	Atrial Fibrillation, n (%)	Previous Stroke, n (%)	Current Smoking, n (%)	Hyperlipidemia, n (%)	DM, n (%)
Dai et al., 2023 (China) (13)	386 (69.05%)	66 (11.81%	Not stated	Not stated	209 (37.38%)	Not stated	144 (25.76%)
Giovanni et al., 2021(Italy) (14)	145 (71.07%)	31 (15.1%)	68 (33.3%)	20 (8.33%)	37 (18.1%)	51 (25%)	21 (10.2%)
Yang et al., 2024 (China) (20)	294 (53.1%)	75 (13.5%)	101 (18.3%)	111 (20.1%)	208 (37.6%)	NS	136 (24,6%)
Zhang et al., 2022 (China) (21)	205 (50.24%)	58 (14.21%)	88 (21.56%)	82 (20.09%)	148 (36.27%)	NS	139 (34.06%)
Wang et al., 2018 (China) (22)	205 (63.86%)	86 (26.79%)	133 (41.43%)	NS	98 (30.53%)	NS	82 (25.54%)
Duan et al., 2023 (China) (23)	353 (61.28%)	125 (21.70%)	113 (19.62%)	121 (21.00%)	210 (36.46%)	115 (19.97%)	230 (39.93%)
Merlino et al., 2024 (Italy) (24)	477 (69.03%)	76 (10.99%)	180 (26.05%)	64 (9.26%)	NS	221 (31.98%)	97 (14.04%)
Chen et al., 2019 (China)(25)	86 (53.1%)	21 (13.1%)	NS	20 (12.5%)	36 (22.5%)	7 (4.4%)	29 (18.1%)
Sun et al., 2023 (China) (26)	269 (63.6%)	NS	221 (52.2%)	NS	NS	NS	59 (13.9%)
Peng et al., 2024 (China) (27)	175 (70%)	36 (14.4%)	50 (20%)	4 (1.6%)	98 (39.2%)	105 (42%)	63 (25.2%)
Shi et al., 2024 (China) (28)	180 (63.2%)	NS	147 (51.6%)	NS	86 (30.2%)	79 (27.7%)	51 (17.9%)
Liu et al., 2022 (China) (29)	547 (74.0%)	NS	334 (45.2%)	152 (20.6%)	NS	NS	234 (31.7%)
Wang et al., 2023 (China) (30)	94 (44.98%)	33 (15.79%)	100 (47.85%)	38 (18.18%)	46 (22.01%)	73 (34.93%)	79 (37.80%)
Peng et al., 2023 (China) (31)	311 (57.4%)	NS	186 (34.3%)	NS	106 (19.6%)	82 (15.1%)	150 (27.7%)
